# Prognostic and Predictive Factors in Patients with Advanced Penile Cancer Receiving Salvage (2nd or Later Line) Systemic Treatment: A Retrospective, Multi-Center Study

**DOI:** 10.3389/fphar.2016.00487

**Published:** 2016-12-20

**Authors:** Carlo Buonerba, Giuseppe Di Lorenzo, Gregory Pond, Giacomo Cartenì, Sarah Scagliarini, Antonio Rozzi, Fernando J. Quevedo, Tanya Dorff, Lucia Nappi, Gaetano Lanzetta, Lance Pagliaro, Bernhard J. Eigl, Gurudatta Naik, Matteo Ferro, Mariano Galdiero, Sabino De Placido, Guru Sonpavde

**Affiliations:** ^1^Department of Clinical Medicine and Surgery, University Federico II of NaplesNaples, Italy; ^2^Istituto Zooprofilattico Sperimentale del MezzogiornoPortici, Italy; ^3^Department of Oncology, McMaster UniversityHamilton, ON, Canada; ^4^Unità Operativa Sperimentazioni Cliniche Oncologia, Azienda Ospedaliera di Rilievo Nazionale ‘Antonio Cardarelli’Naples, Italy; ^5^Dipartimento di Oncologia, Istituto Neurotraumatologico ItalianoGrottaferrata, Italy; ^6^Division of Medical Oncology, Department of Oncology, Mayo Clinic College of MedicineRochester, MN, USA; ^7^Norris Comprehensive Cancer Center, University of Southern California Keck School of MedicineLos Angeles, CA, USA; ^8^Division of Medical Oncology, British Columbia Cancer Agency, Vancouver Cancer Center, University of British ColumbiaVancouver, BC, Canada; ^9^Department of Urologic Sciences, Vancouver Prostate Centre, University of British ColumbiaVancouver, BC, Canada; ^10^Section of Hematology-Oncology, Department of Medicine, University of Alabama at Birmingham Comprehensive Cancer CenterBirmingham, AL, USA; ^11^Department of Urology, European Institute of OncologyMilan, Italy; ^12^Ios and Coleman Medicina Futura Medical CenterNaples, Italy

**Keywords:** penile squamous cell carcinoma, salvage, prognosis, classification

## Abstract

**Introduction and objectives:** Metastatic penile squamous cell carcinoma (PSCC) is associated with dismal outcomes with median overall survival (OS) of 6–12 months in the first-line and <6 months in the salvage setting. Given the rarity of this disease, randomized trials are difficult. Prognostic risk models may assist in rational drug development by comparing observed outcomes in nonrandomized phase II studies and retrospective data vs. predicted outcomes based on baseline prognostic factors in the context of historically used agents. In this retrospective study, we constructed a prognostic model in the salvage setting of PSCC patients receiving second or later line systemic treatment, and also explored differences in outcomes based on type of treatment.

**Materials and methods**: We performed a chart review to identify patients with locally advanced unresectable or metastatic PSCC who received second or later line systemic treatment in centers from North America and Europe. The primary outcome was OS from initiation of treatment, with secondary outcomes being progression-free survival (PFS) and response rate (RR). OS was estimated using the Kaplan-Meier method. Cox proportional hazards regression was used to identify prognostic factors for outcomes using univariable and multivariable models.

**Results:** Sixty-five patients were eligible. Seventeen of 63 evaluable patients had a response (27.0%, 95% confidence interval [CI] = 16.6–39.7%) and median OS and PFS were 20 (95% CI = 20–21) and 12 (95% CI = 12, 16) weeks, respectively. Visceral metastasis (VM) and hemoglobin (Hb) ≤ 10 gm/dl were consistently significant poor prognostic factors for both OS and PFS, and Hb was also prognostic for response. The 28 patients with neither risk factor had a median OS (95% CI) of 24 (20–40) weeks and 1-year (95% CI) OS of 13.7% (4.4–42.7%), while the 37 patients with 1 or 2 risk factors had median OS (95% CI) of 20 (16–20) weeks and 1-year (95% CI) OS of 6.7% (1.8–24.9%). Cetuximab-including regimens were associated with a trend for improved RR compared to other agents (Odds ratio = 5.05, 95% CI = 0.84–30.37, *p* = 0.077). Taxanes vs. non-taxane, and combination vs. single agent therapy was not associated with improved outcomes. The study is limited by its modest sample size.

**Conclusion:** This is the first prognostic classification proposed for patients receiving salvage systemic therapy for advanced PSCC. The presence of VM and Hb ≤ 10 gm/dl was associated with poor OS and PFS. Cetuximab appeared to be associated with better RR. This prognostic model may assist in salvage therapy drug development for this orphan disease by improving interpretation of outcomes seen in nonrandomized data.

## Introduction

In developed countries, penile squamous cell carcinoma (PSCC) is relatively rare, but less developed countries exhibit higher incidences. In 2016, approximately 2000 new cases and 300 deaths from penile cancers were predicted to occur in the United States (Sonpavde et al., 2013; Siegel et al., 2016). Although most patients present with localized disease, locoregional unresectable, or metastatic relapses are common after radical treatment based on surgery and/or radiotherapy. PSCC patients with metastatic disease have a poor prognosis, with a median overall survival (OS) in the range of 6–12 months using platinum-based combination chemotherapy in published retrospective studies (Theodore et al., 2008; Di Lorenzo et al., 2012; Zhang et al., 2015). Patients with progressive disease following prior chemotherapy have dismal outcomes with OS <6 months (Di Lorenzo et al., 2011).

Prognostic nomograms have been reported in patients with localized disease undergoing surgery (Kattan et al., 2006) and also in patients with metastatic disease receiving first-line systemic treatment (Pond et al., 2014), Unfortunately, there is a lack of such prognostic classifications for patients receiving second or later-line salvage systemic treatment for advanced penile cancer, i.e., locally advanced unresectable or metastatic disease. Prognostic classifications/models may be useful for the interpretation of phase II trial outcomes, especially in the particular case of penile carcinoma, since the rarity of the disease makes it difficult to conduct large, randomized-controlled trials. Moreover, salvage regimens may be tailored for the patient population, e.g., those with particularly dismal outcomes may warrant the evaluation of more aggressive combination regimens.

This retrospective review was therefore conducted with an aim of identifying prognostic factors for patient outcomes, notably survival, amongst patients with PSCC receiving salvage systemic therapy. After identifying prognostic factors, it was desired to construct a prognostic model which could be used for risk stratifying patients. In addition, a secondary aim of this study was to explore for differences between salvage treatments, in particular anti-epidermal growth factor receptor (EGFR) containing vs. non anti-EGFR containing therapy.

## Materials and methods

### Patient population

A retrospective chart review was performed in order to identify patients with locally advanced unresectable or metastatic PSCC who received second or later lines of systemic treatment in referral medical centers from North America, Europe, and Japan. Eligible patients were men receiving systemic treatment (but not concurrent chemo-radiotherapy or adjuvant treatment) for locally advanced unresectable or metastatic disease or neoadjuvant therapy preceding definitive local therapy after being treated with ≥1 systemic treatments for locally advanced unresectable or metastatic disease or neoadjuvant therapy preceding definitive local therapy. Patients who received adjuvant therapy following local definitive therapy and concurrent systemic therapy and radiation as the only prior systemic treatment were excluded.

The following patient and disease characteristics at baseline were requested: Eastern Cooperative Oncology Group (ECOG) performance status (PS), American Joint Committee on Cancer 7th Edition clinical stage, sites of metastases (soft tissue vs. visceral), hemoglobin, race, smoking status, circumcision status, peripheral blood neutrophil count, peripheral blood lymphocyte count, albumin, family history of penile cancer, history of precancerous lesion, human immunodeficiency virus (HIV) status, lymphovascular invasion (LVI), histologic subtype, systemic therapy regimen, postchemotherapy surgery received, time from last prior therapy, number of previous lines received (measured including biologic and chemotherapy ± radiation), time to objective tumor progression, time to last follow-up, and radiological best response.

### Statistical methods

Summary statistics were used to describe the patient, tumor, and treatment characteristics, as well as outcomes. The primary outcome was overall survival, with secondary outcomes being progression-free survival and response rate. Descriptive statistics were used to summarize patient and tumor information, as well as outcomes. Survival was defined as the time from initiation of salvage systemic treatment until death due to any cause, while progression-free survival (PFS) was defined as the time from initiation of salvage systemic treatment until disease progression (clinical or radiological) as defined in the patients chart. Any patient without death or progression event was censored at the last chart entry. Response was defined using the RECIST criteria (Therasse et al., 2000).

The Kaplan-Meier method was used to estimate time to event outcomes (OS and PFS). Cox proportional hazards regression and logistic regression were used to explore factors potentially prognostic of time to event outcomes and response respectively. Each factor was examined in univariate models. Stepwise selection was initially used for constructing a multivariable model, with backward and forward selection processes used to explore the robustness of this model. Owing to the modest sample size, clinical and statistical expertise was then used to create a final prognostic model.

Some factors with little variability (e.g., family history, HIV status) along with factors having a lot of missing data or unknown information (e.g., HPV status) were excluded from being a potential candidate in the multivariate model. Transformations (e.g., using a logarithmic transformation for non-normal data) or categorization (e.g., number of prior lines of treatment) was also performed for statistical purposes. All tests were two-sided and statistical significance was defined as a *p*-value of 0.05 or less throughout.

This retrospective review study was conducted according to the existing privacy laws by using anonymized records and according to the Declaration of Helsinki. The protocol was reviewed and approved by the institutional review board/independent ethics committee of the Institutions that requested to do so according to local laws and policies.

## Results

### Patient characteristics and treatment outcomes

Sixty-five patients treated at 8 participating Institutions were available for the analysis (Table 1). The median OS was 20 (95% CI = 20–21) weeks, while the median PFS was 12 (95% CI = 12–16) weeks (Table 2). Prior cisplatin had been administered to 52 patients (80%) and most patients had stage 4 disease (*N* = 53, 81.5%), with visceral disease in 30 patients (46.2%). Treatment regimens administered included a taxane agent in 48 patients (73.8%) and cetuximab in 17 patients (26.2%). Five patients received either bleomycin/methotrexate/cisplatin, single agent capecitabine, cisplatin/5FU, gemcitabine/navelbine, or Methotrexate/bleomycin, respectively, while single-agent gemcitabine was administered to 4 patients. Most patients had ECOG-PS 0-1 (*N* = 52, 82.6%) and were of Caucasian race (*N* = 59, 90.8%). Seventeen of 63 evaluable patients had a response (27.0%, 95% confidence interval [CI] = 16.6–39.7%).

**Table 1:** **Patients' characteristics**.

**Characteristic**	**Statistic**	***N***	**Result**
Age	Mean (std dev)	65	61.3 (8.7)
Hemoglobin, g/dL	Mean (std dev)	64	11.7 (1.6)
Neutrophils, /mm^3^	Median (range)	63	4000 (2100, 21000)
Lymphocytes, /mm^3^	Median (range)	62	1055 (400, 2100)
Neutrophils-Lymphocytes Ratio	Median (range)	62	3.8 (1.6, 17.5)
	≥5		21 (33.9)
Albumin, g/dL	Mean (std dev)	60	3.47 (0.54)
Time from prior treatment, weeks	Median (range)	64	20 (4, 176)
Smoking status	Never	64	16 (25.0)
	Past smoker		29 (45.3)
	Current smoker		19 (29.7)
Circumcision	No	60	49 (81.7)
	Yes, Postnatal		8 (13.3)
	Yes, Neonatal		3 (5.0)
ECOG status	0	63	26 (41.3)
	1		26 (41.3)
			11 (17.5)
	2		
Race	Caucasian	65	59 (90.8)
	Hispanic/Latin American		6 (9.2)
Family history	None	65	63 (96.9)
	Other than 1st degree relative		2 (3.1)
Prior history of precancerous lesion	Yes	65	22 (33.9)
HPV status	Yes	33	18 (54.5)
HIV status	Yes	63	1 (1.6)
Stage	IV	65	53 (81.5)
Visceral disease	Yes	65	30 (46.2)
Lymph-vascular invasion	Yes	14	11 (78.6)
Prior cisplatin-based chemotherapy	Yes	65	52 (80.0)
Prior lines of treatment	1	65	37 (56.9)
	2		8 (12.3)
	3		20 (30.8)
Agent	Paclitaxel/carboplatin		1
	Docetaxel		3
	Paclitaxel		30
	Paclitaxel/ifosfamide/cisplatin		1
	Bleomycin/methotrexate/cisplatin		1
	Capecitabine		1
	Cetuximab		8
	Cetuximab/docetaxel		6
	Cisplatin/5FU		1
	Gemcitabine		4
	Gemcitabine/navelbine		1
	Methotrexate/bleomycin		1
	Paclitaxel/5FU		2
	Paclitaxel/carboplatin/cetuximab		1
	Paclitaxel/cetuximab		2
	Paclitaxel/cisplatin/gemcitabine		1
	Includes Cetuximab		17 (26.2)
	Includes a taxane		48

**Table 2:** **Patients' outcomes**.

**Outcomes**			
Best response (RECIST)	Complete Response	63	2 (3.2)
	Partial Response		15 (23.8)
	Stable Disease		16 (25.4)
	Progressive Disease		30 (47.6)
Progression-free survival	N (%) Events	64	62 (96.9)
	Median (95% CI) Weeks		12 (12, 16)
	6-month (95% CI) PFS		10.9 (4.8, 19.9)
	1-year (95% CI) PFS		3.6 (0.7, 10.9)
Overall survival	N (%) Deaths	65	55 (84.6)
	Median (95% CI) Weeks		20 (20, 21)
	6-month (95% CI) OS		28.2 (17.5, 39.9)
	1-year (95% CI) OS		9.9 (3.5, 20.1)

### Univariable analyses examining association of variables with outcomes

Tables 3–5 show the univariable results from Cox and logistic regression models assessing the prognostic ability of factors for OS, PFS and response respectively. Age (hazard ratio [HR]/decade = 1.48, 95% CI = 1.09–2.00, *p* = 0.011), low hemoglobin (HR = 0.76, 95% CI = 0.62–0.95, *p* = 0.014), low lymphocytes (HR/100 mm3 = 0.91, 95% CI = 0.84–0.98, *p* = 0.015), and prior non-cisplatin-based chemotherapy (HR = 2.08, 95% CI = 1.08–4.17, *p* = 0.029) were all significantly prognostic for poor OS in univariable models (Table 3). Age, anemia and visceral disease were also significantly associated with poor PFS on univariable analyses (Table 4). Higher hemoglobin and albumin levels, better ECOG-PS and cetuximab were associated with better odds of response to systemic treatment (Table 5).

**Table 3:** **Prognostic factors of overall survival**.

**Characteristic**	**Units**	**Hazard ratio (95% CI)**	***p*****-value**
Age	per decade	1.48 (1.09, 2.00)	0.011
Hemoglobin, g/dL	per g/dL	0.76 (0.62, 0.95)	0.014
Neutrophils, /mm^3^	Log-transformed	0.90 (0.53, 1.52)	0.69
Lymphocytes, /mm^3^	per 100 mm^3^	0.91 (0.84, 0.98)	0.015
Neutrophils-Lymphocytes Ratio	≥5 vs. <5	0.81 (0.45, 1.45)	0.47
Albumin, g/dL	per g/dL	0.70 (0.38, 1.26)	0.23
Time from prior treatment, weeks	Log-transformed	1.08 (0.75, 1.54)	0.69
Smoking status	Never	0.98 (0.46, 2.10)	0.11
	Past Smoker	1.78 (0.92, 3.41)	
	Current Smoker	REFERENCE	
Circumcision	Yes vs. No	1.31 (0.65, 2.64)	0.45
ECOG status	0	0.75 (0.34, 1.63)	0.76
	1	0.85 (0.39, 1.85)	
	2	REFERENCE	
Race	Caucasian vs. Hispanic	0.91 (0.35, 2.34)	0.84
Family history	Other vs. None	0.41 (0.10, 1.70)	0.22
Prior history of precancerous lesion	Yes vs. No	1.33 (0.76, 2.33)	0.31
HPV status	Yes vs. No	1.48 (0.72, 3.03)	0.29
HIV status	Yes vs. No	1.23 (0.17, 8.99)	0.84
Stage	IV vs. III	1.90 (0.89, 4.06)	0.096
Visceral disease	Yes vs. No	1.63 (0.95, 2.80)	0.076
Lymph-vascular invasion	Yes vs. No	0.73 (0.14, 3.67)	0.70
Prior cisplatin-based chemotherapy	Yes vs. No	0.48 (0.24, 0.93)	0.029
Prior lines of treatment	≥2 vs. 1	0.93 (0.54, 1.61)	0.80
Cetuximab	Yes vs. No	1.15 (0.63, 2.09)	0.65

**Table 4:** **Predictive factors of progression-free survival**.

**Characteristic**	**Units**	**Hazard ratio (95% CI)**	***p*****-value**
Age	per decade	1.39 (1.02, 1.89)	0.035
Hemoglobin, g/dL	per g/dL	0.82 (0.68, 0.99)	0.041
Neutrophils, /mm^3^	Log-transformed	1.21 (0.75, 1.94)	0.43
Lymphocytes, /mm^3^	per 100 mm^3^	0.99 (0.92, 1.06)	0.70
Neutrophils-lymphocytes ratio	≥5 vs. <5	0.80 (0.47, 1.38)	0.43
Albumin, g/dL	per g/dL	0.60 (0.33, 1.09)	0.092
Time from prior treatment, weeks	Log-transformed	1.02 (0.72, 1.44)	0.92
Smoking status	Never	1.16 (0.57, 2.35)	0.26
	Past Smoker	1.63 (0.87, 3.05)	
	Current Smoker	REFERENCE	
Circumcision	Yes vs. No	1.07 (0.55, 2.08)	0.83
ECOG status	0	0.76 (0.37, 1.57)	0.74
	1	0.88 (0.43, 1.80)	
	2	REFERENCE	
Race	Caucasian vs. Hispanic	0.51 (0.21, 1.19)	0.12
Family history	Other vs None	0.23 (0.05, 1.02)	0.054
Prior history of precancerous lesion	Yes vs. No	1.12 (0.66, 1.89)	0.67
HPV status	Yes vs. No	1.47 (0.70, 3.09)	0.31
HIV status	Yes vs. No	0.70 (0.10, 5.09)	0.72
Stage	IV vs. III	1.28 (0.68, 2.42)	0.45
Visceral disease	Yes vs. No	1.77 (1.06, 2.95)	0.030
Lymph-vascular invasion	Yes vs. No	0.54 (0.13, 2.24)	0.39
Prior cisplatin-based chemotherapy	Yes vs. No	0.96 (0.51, 1.81)	0.90
Prior lines of treatment	≥2 vs. 1	1.04 (0.62, 1.72)	0.90
Cetuximab	Yes vs. No	0.95 (0.54, 1.67)	0.86

**Table 5:** **Predictive factors of response**.

**Characteristic**	**Units**	**Odds ratio (95% CI)**	***p*****-value**
Age	per decade	0.67 (0.35, 1.28)	0.23
Hemoglobin, g/dL	per g/dL	2.43 (1.37, 4.29)	0.002
Neutrophils, /mm^3^	Log-transformed	0.31 (0.08, 1.14)	0.078
Lymphocytes, /mm^3^	per 100 mm^3^	0.98 (0.83, 1.16)	0.83
Neutrophils-lymphocytes ratio	≥5 vs. <5	3.36 (0.84, 13.44)	0.087
Albumin, g/dL	per g/dL	4.82 (1.39, 16.72)	0.013
Time from prior treatment, weeks	Log-transformed	0.80 (0.36, 1.79)	0.59
Smoking status	Never	4.50 (0.75, 26.92)	0.24
	Past Smoker	3.37 (0.63, 17.96)	
	Current Smoker	REFERENCE	
Circumcision	Yes vs. No	1.72 (0.43, 6.89)	0.45
ECOG status	0	7.86 (0.87, 71.06)	0.036
	1	1.82 (0.18, 18.41)	
	2	REFERENCE	
Race	Caucasian vs. Hispanic	1.95 (0.21, 18.03)	0.56
Family history	Other vs. None	0.36 (0.02, 6.02)	0.47
Prior history of precancerous lesion	Yes vs. No	0.48 (0.14, 1.70)	0.25
HPV status	Yes vs. No	2.00 (0.40, 9.91)	0.40
HIV status	Yes vs. No	Not Calculable	–
Stage	IV vs. III	1.14 (0.27, 4.81)	0.86
Visceral disease	Yes vs. No	0.97 (0.32, 2.96)	0.96
Lymph-vascular invasion	Yes vs. No	Not Calculable	–
Prior cisplatin-based chemotherapy	Yes vs. No	2.08 (0.41, 10.67)	0.38
Prior lines of treatment	≥2 vs. 1	0.65 (0.21, 2.05)	0.46
Cetuximab-containing regimen	Yes vs. No	3.65 (1.10, 12.12)	0.034

### Multivariable analyses examining association of variables with clinical outcomes

Visceral disease (HR = 1.56, 95% CI = 0.90–2.70, *p* = 0.11) and number of lymphocytes (HR/100 mm^3^ = 0.91, 95% CI = 0.84–0.99, *p* = 0.023) were the variables retained in the model after the stepwise selection. Visceral disease (HR = 1.77, 95% CI = 1.06–2.95, *p* = 0.030) was the only prognostic factor of poor PFS. Higher hemoglobin levels (OR = 2.75 per each g/dL increase, 95% CI = 1.44–5.25, *p* = 0.002) and cetuximab-containing treatment regimen (OR = 5.86 for cetuximab-containing regimen versus no cetuximab, 95% CI = 1.35–25.47, *p* = 0.019) were prognostic factors for response.

Based on the results of the stepwise selection along with clinical and statistical expertise, a 3-factor model, including anemia status, visceral disease status and lymphocytes, was proposed. However, after adjusting for the other 2 factors, number of lymphocytes was deemed to not add statistically important information as a prognostic factor for OS (*p* = 0.076), PFS (*p* = 0.78) nor response (*p* = 0.082). Thus, a two-factor prognostic model for OS was ultimately proposed. Amongst these patients, having visceral disease (HR = 2.06, 95% CI = 1.18–3.59, *p* = 0.011) was associated with worse prognosis, while higher hemoglobin levels were associated with decreased risk of death (HR = 0.73 per g/dL, 95% CI = 0.58–0.91, *p* = 0.006). Similar results were observed for PFS, with visceral disease being associated with increased risk of progression (HR = 2.01, 95% CI = 1.18–3.43, *p* = 0.010) and higher hemoglobin levels being associated with decreased risk of progression (HR = 0.78 per each g/dL increase, 95% CI = 0.62–0.95, *p* = 0.015). Higher hemoglobin levels were also associated with increased odds of having a radiologic response (OR = 2.45, 95% CI = 1.38–4.36, *p* = 0.002).

### Prognostic stratification

**Figure 1:**
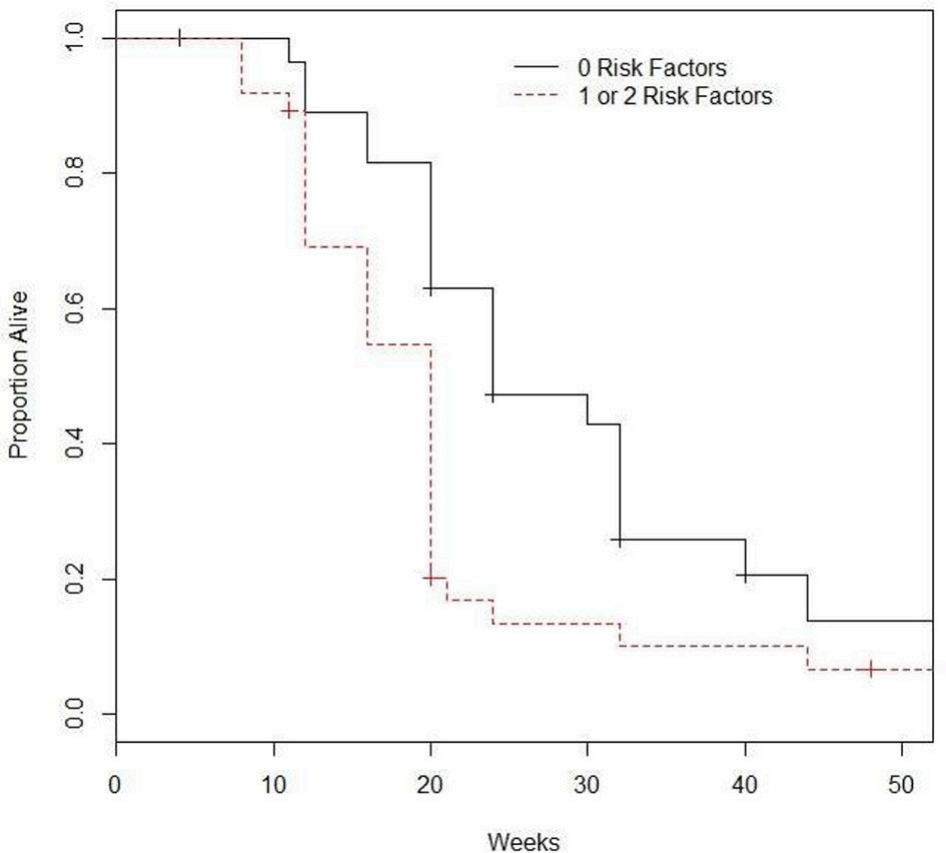
Survival based on number of prognostic factors^*^. ^*^Prognostic factors included are visceral metastasis and hemoglobin ≤10.

Patients were considered as having a risk factor if they had visceral disease or hemoglobin levels Hb ≤ 10 g/dL). There were 28, 33, and 4 patients with 0, 1, and 2 risk factors. Given the small sample size, patients with 1 and 2 risk factors were combined. The 28 patients with neither risk factor had a median OS of 24 (95% CI: 20–40) weeks, and 6-month and 1-year OS of 47.2% (95% CI: 31.5–70.8%) and 13.7% (4.4–42.7%), see Figure 1. In comparison, the 37 patients with 1 or 2 risk factors had median OS of 20 (95% CI: 16–20) weeks, 6-month and 1-year OS of 13.4% (95% CI: 5.6–32.1%) and 6.7% (95%: 1.8–24.9%). The difference did achieve statistical significance (*p* = 0.0049). Of evaluable patients with 0 and 1–2 risk factors, 9/26 (34.6%) and 8/37 (21.6%) had a response.

## Discussion

In this multi-institutional retrospective study cohort, systemic therapy after failure of primary cisplatin-based therapy was associated with limited disease control. Median PFS and OS were 12 and 20 weeks, respectively, with about 90% of patients progressing and dying within 6 months and 1 year, respectively. Our results are consistent with those recently published in a smaller study by Wang et al. that analyzed outcomes of 19 patients treated with local or systemic therapy for recurrent PSCC after neoadjuvant paclitaxel, ifosfamide, and cisplatin chemotherapy (Wang et al., 2015). We also constructed a prognostic model including visceral disease status and Hb level which discriminated patients into statistically significantly different groups. However, our study is limited by small sample size of only 65 patients. Some candidates for prognostic factors such as stage, ECOG-PS, albumin, HPV status and number of prior regimens were not independently prognostic, possibly due to the modest sample size.

It is noteworthy that in our retrospective study cohort, the 95% CI interval for OS was 20–21 weeks, which implies that the prognosis of our cohort population was homogeneously poor. The negative prognostic value of visceral disease observed in this study was consistent with a previously published retrospective analysis of 140 patients with penile squamous cell carcinoma receiving first-line systemic therapy, which showed visceral disease had an increased hazard ratio for death of 2.42 (95% CI: 1.47–4.00) (Pond et al., 2014). Also, in a study including 26 patients treated with concurrent chemo-radiotherapy for advanced penile cancer, a trend for a detrimental prognostic effect of visceral disease was reported (HR = 3.35; 95% CI: 0.69–16.21; *P* = 0.13). Indeed, a prognostic classification recently proposed in the first-line setting of advanced PSCC receiving cisplatin-based chemotherapy included one clinical factor, visceral disease, and one tumor tissue molecular factor, MAML2 gene expression (Necchi et al., 2016a).

Incorporating clinical judgment, we proposed a two-variable based risk group classification based on VM (absent vs. present) and baseline hemoglobin levels (≤10 vs. >10). These factors may provide a simple prognostic classification tool useful for clinical practice and for patient selection/stratification criteria for research purposes, pending their external validation.

Of note, our study cohort included 17 patients treated with cetuximab, which was administered either alone (8 patients) or in combination with a taxane/platinum agent (9 patients). Evidence of activity of anti-EGFR agents in advanced penile cancer was originally reported when administered as single agent (Necchi et al., 2016b) or in combination with chemotherapy (Rescigno et al., 2012). In a literature review of individual data of 28 patients with advanced penile cancer receiving anti-EGFR monoclonal antibodies (cetuximab, panitumumab, and nimotuzumab), a 50% radiological response rate was achieved, but a median PFS of only approximately 3 months (interquartile range, 1.5–5.78, Di Lorenzo et al., 2015). From a biological perspective, EGFR appears a promising target in penile cancer, as it is universally expressed and frequently phosphorylated (Di Lorenzo et al., 2013a), but infrequently amplified (Di Lorenzo et al., 2013a), or mutated (Di Lorenzo et al., 2013b), while mutations of downstream signaling proteins (such as KRAS/BRAF) are rare (Gou et al., 2013). Nevertheless, we failed to identify a signal of prolonged OS associated with the use of cetuximab in our study cohort. The use of anti-EGFR agents may be most beneficial in patients requiring tumor shrinking (e.g., symptomatic metastatic patients or patients receiving neoadjuvant treatment, Luo et al., 2015). Ongoing phase II trials are evaluating dacomitinib and afatinib, oral pan-Her inhibitors, as neoadjuvant or salvage therapy for PSCC.

Finally, the association of a higher baseline lymphocyte count with improved OS outcomes is intriguing, although the final proposed model does not include this variable. Kasuga et al. (2016) found that a higher baseline neutrophil to lymphocyte ratio was associated with increased disease specific mortality in patients with penile cancer. Interestingly, increased lymphocyte baseline count was associated with improved prognosis in multiple malignancies, including melanoma patients treated with pembrolizumab, an anti-programmed death (PD)-1 immunotherapeutic agent (Weide et al., 2016). On the grounds of the association of PD-L1 tumor expression with response to anti-PD-1 agents in solid malignancies (melanoma, non-small cell lung cancer) (Di Lorenzo et al., 2016) and of PD-L-1 expression in penile cancer patients (Udager et al., 2016), pembrolizumab will be tested in a single-arm phase II study (ClinicalTrials.gov Identifier: NCT02837042) in PSCC patients. In such a population of PSCC treated with immunotherapy, the prognostic value of baseline lymphocyte count may be clinically relevant.

To summarize, we have proposed the first prognostic classification for patients receiving salvage systemic therapy for advanced PSCC, which can enhance interpretation of outcomes seen in nonrandomized salvage therapy data. The presence of visceral disease and Hb ≤ 10 gm/dl was associated with poor OS and PFS. A signal for incremental survival benefit with cetuximab was not confirmed in spite of an apparently improved response rate. This prognostic model is a first step toward prognostic classification in this setting. Analysis of a larger dataset incorporating clinical and molecular factors may further optimize prognostic classification for this orphan disease and provide insights regarding potential therapeutic targets.

## Author contributions

All authors listed approved the article for publication and have made substantial, direct and intellectual contribution to the work, as detailed below. Study concept and design: CB, GD, GS, and GP; Acquisition of data: SS, AR, FQ, TD, LN, GL, LP, BE, GN, MF, and MG; Analysis and interpretation of data: All authors; Drafting of the manuscript: CB, GD, GS, and GP; Critical revision of the manuscript for important intellectual content: All authors; Statistical analysis: GP.

### Conflict of interest statement

Guru Sonpavde: Consultant for Bayer, Sanofi, Pfizer, Novartis, Eisai, Janssen, Amgen, Astrazeneca, Merck, Genentech, Argos, Agensys; Research support to institution from Bayer, Onyx, Celgene, Boehringer-Ingelheim, Merck, Pfizer; Author for Uptodate; Speaker for Clinical Care Options. Bernhard J. Eigl: Honoraria from Roche, AstraZeneca, Merck, Pfizer. Grant support from Roche. Tanya Dorff: Consulting for Genentech, dendreon, Pfizer, bayer. Speaker for exelixis and astellas. Lance Pagliaro: Honoraria—Sanofi and Novartis. The other authors declare that the research was conducted in the absence of any commercial or financial relationships that could be construed as a potential conflict of interest.
